# The role of trust and health literacy in nurse-delivered point-of-care STI testing for pregnant women living with HIV, Tshwane District, South Africa

**DOI:** 10.1186/s12889-020-08689-3

**Published:** 2020-04-28

**Authors:** Andrew Medina-Marino, Katherine Glockner, Emily Grew, Lindsey De Vos, Dawie Olivier, Jeffrey Klausner, Joseph Daniels

**Affiliations:** 1 Fundation for Professional Development, 10 Rochester Road, East London, 5217 South Africa; 2 The Desmond Tutu HIV Centre, University of Cape Town, Cape Town, South Africa; 3 Mailman School of Public Health, Columbia University, New York, NY USA; 4 Northeastern University, Boston, MA USA; 5 David Geffen School of Medicine and Fielding School of Public Health, UCLA, Los Angeles, CA USA; 6 Charles R. Drew University of Medicine and Science, Los Angeles, CA USA

**Keywords:** Sexually transmitted infections, Point-of-care testing, Patient-provider communications, Pregnancy, HIV, South Africa

## Abstract

**Background:**

Sexually transmitted infections (STIs) during pregnancy result in neonatal morbidity and mortality, and may increase mother-to-child-transmission of HIV. Yet the World Health Organization’s current syndromic management guidelines for STIs leaves most pregnant women undiagnosed and untreated. Point-of-care (POC) diagnostic tests for STIs can drastically improve detection and treatment. Though acceptable and feasible, poor medication adherence and re-infection due to lack of partner treatment threaten the programmatic effectiveness of POC diagnostic programmes.

**Methods:**

To engender patient-provider trust, and improve medication adherence and disclosure of STI status to sexual partners, we trained study nurses in compassionate care, good clinical practices and motivational interviewing. Using qualitative methods, we explored the role patient-provider communications may play in supporting treatment adherence and STI disclosure to sexual partners. Nurses were provided training in motivational interviewing, compassionate care and good clinical practices. Participants were interviewed using a semi-structured protocol, with domains including STI testing experience, patient-provider communication, and HIV and STI disclosure. Interviews were audio-recorded, transcribed and analyzed using a constant comparison approach.

**Results:**

Twenty-eight participants treated for *Chlamydia trachomatis* (CT), *Trichomonas vaginalis* (TV), and/or *Neisseria gonorrhea* (NG) were interviewed. Participants described strong communications and trusting relationships with nurses trained in patient-centered care training and implementing POC STI diagnostic testing. However, women described a delayed trust in treatment until their symptoms resolved. Women expressed a limited recall of their exact diagnosis, which impacted their ability to fully disclose their STI status to sexual partners.

**Conclusions:**

We recommend implementing patient health literacy programmes as part of POC services to support women in remembering and disclosing their specific STI diagnosis to sexual partners, which may facilitate partner treatment uptake and thus decrease the risk of re-infection.

## Background

In 2017, South Africa reported that 30.8% of women attending antenatal care (ANC) services were living with HIV [1]. These findings emphasize the need to ensure optimal implementation of prevention of mother to child transmission (PMTCT) programs, including early and immediate access to antiretroviral therapy (ART) [2, 3]. Sexually transmitted infections (STI) during pregnancy may increase MTCT of HIV [4, 5]. A recent study from Tshwane District, South Africa, reported that 47.8% of pregnant women living with HIV attending their first ANC visit for their current pregnancy tested positive for an STI, of which nearly 60% were asymptomatic [2]. These results continue to suggest that syndromic management for STIs, especially during pregnancy, is insufficient to optimally detect and treatment these infections.

As point-of-care (POC) technologies for STI diagnosis become available, especially in resource-poor health systems, pregnant women must be provided access to testing services. Recent work to integrate STI molecular diagnostic testing into ANC services for pregnant women living with HIV was shown to be acceptable and feasible [3]. However, to ensure optimal treatment outcomes, patients must be counselled to complete their course of treatment. Moreover, to reduce the risk of re-infection, patients must be empowered and supported to disclose their STI status to their partner(s), and the health system must provide optimal services for partner treatment uptake [6–8]. Implementing a comprehensive package of services (i.e., diagnostic testing, medication adherence counselling, STI status disclosure support and partner treatment uptake) will certainly improve sexual, reproductive and maternal-child health outcomes [3, 9]. However, to ensure optimal clinical outcomes of HIV and STI services, effective patient-provider communication must engender patient trust and engagement in care [10–14]. Unfortunately, challenges that affect patient-provider communication include women’s internalized shame and disease-associated stigma that limits their access to care [15]. It has also been shown that patients experience stigma and discrimination from nurses, especially with regard to STI testing and treatment, which can reinforce STI stigma during pregnancy [16, 17]. As a result, women living with HIV and diagnosed with an STI report lower rates of disclosure to their sexual partners [18, 19].

In South Africa, nurse training in patient-provider communication is limited, as clinical care is generally prescriptive with few opportunities to engage in conversations about specific diagnosis and treatment plans [20]. Although nurse providers can build relationships with patients over time, in many African settings patients do not see the same nurse at subsequent clinic visits. Consequently, this can compromise a patient’s ability to understand their diagnosis and treatment, leading to high rates of non-adherence and reluctance to access care [21]. While patient-provider communications can directly impact patient outcomes, so too can a patient’s health literacy [22–24]. For example, lack of basic health knowledge may limit a patient’s understanding of the importance of daily adherence and disclosing diagnoses to partners [21]. Research has also shown that disclosure of HIV or STI status is important to PMTCT programming as involving male partners can reduce sexual risk, improve adherence, and facilitate communication between partners [25, 26]. Moreover, disclosure to partners has been shown to facilitate partner testing and treatment uptake [27]. Given that STIs often include risks of reinfection with continued exposure, any opportunity to increase partners' treatment may directly impact sexual and reproductive health, and in the context of pregnancy, may directly reduce risk of infant morbidity and mortality [28–30].

As part of a larger study to assess the acceptability and feasibility of implementing comprehensive POC STI diagnostic testing, same day treatment and partner treatment services into antenatal care services, we trained study nurses in compassionate care, good clinical practices and motivational interviewing. This was done to engender patient-provider trust, improve medication adherence, and support disclosure of women’s STI status to sexual partners. In this sub-study we qualitatively explored the role patient-provider communications played in supporting treatment adherence and STI disclosure to sexual partners, with the goal of informing and improving future services and support as part of comprehensive POC STI testing services.

## Methods

We nested a qualitative sub-study within a larger, previously described cohort study aimed at integrating STI diagnostic testing into ANC services [2, 3]. Briefly, in the larger study pregnant women living with HIV were offered diagnostic testing via GeneXpert® (Sunnyvale, California, U.S.A.) for *Chlamydia trachomatis* (CT), *Neisseria gonorrhoeae* (NG), and *Trichomonas vaginalis* (TV); in South Africa, syndromic management is standard care [31]. Women with a positive STI test result were provided targeted treatment and asked to return to the clinic 3 weeks post-treatment for re-testing (i.e., test-of-cure, TOC) and additional treatment if necessary. As part of the STI testing intervention package, at the time of diagnosis study nurses explicitly provided the clinical and local vernacular names of the STI(s) the participant tested positive for, the health consequences to her, her unborn child and sexual partner(s) if the STI(s) remained untreated. Nurses also provided support for disclosing their STI status to sexual partners, including disclosure counselling and engaging in conversation about how a participant may approach a conversation with their sexual partner(s). Regarding partner treatment, women were provided an option to take to their partners either a treatment pill packet or a referral letter for STI clinical services. This qualitative sub-study aimed to assess the experiences of pregnant women living with HIV with nurses providing POC STI diagnostic testing with same day treatment.

### Research nurse training

All research nurses completed a two-week training in the study procedures, South African guidelines for STI management and maternity care, and Good Clinical Practice that integrated clinical ethics case studies. Included in this two-week training, research nurses also completed 10 h of training in compassionate care and motivational interviewing skills as it applied to STI disclosure and referral counseling [32], as well as training in motivational interviewing that is not part of standard nurse educational curriculum in South Africa. Practice-based approaches were integrated into study training and preparation activities. This allowed nurses to build upon their clinical experiences while learning how to apply their new skills during counseling simulations prior to initiating study enrollment procedures. Through this training, the objective was to develop nurse skills in building a clinical relationship with study participants, and nearly all nurses saw the same participants throughout the study. Additional trainings were provided throughout study period to ensure that the research nurses were updated on study activity changes including those informed by any clinical cases that were discussed with the study clinician.

### Participant recruitment

Participant recruitment has been previously described for the larger study [8]. For the qualitative sub-study, participants also had to: 1) test positive for CT, NG, and/or TV at their first ANC visit, 2) return to the clinic for a test-of-cure, and 3) provide informed consent to participate in a qualitative in-depth interview (IDI). Upon providing informed consent for the IDI, participants were interviewed in a private space within or outside the clinic by trained study staff.

### Data collection

IDIs were conducted using a semi-structured protocol that covered the domains of testing and nurse experiences, partner communication, STI disclosure to partners and their response, partner treatment uptake, relationship decision-making, HIV disclosure and financial independence. Interviews were conducted in the participants preferred language (i.e., English, seTswana or sePedi), with interviews lasting between 35 and 90 min [33]. All IDIs were audio-recorded, translated and transcribed with quality checks conducted by an independent researcher.

### Data analysis

Five researchers open-coded seven transcripts and developed a codebook to be used for all transcripts. All codes indicated either preventative or risk behaviors. Two researchers then coded the remaining transcripts following a random assignment process. Quality assurance procedures were applied to ensure that coding was correctly utilized and documented. The research team utilized a constant comparison approach to analyze data. Themes were developed over time, with a focus on how the relationship between the nurse and participant developed during the study [34]. Our approach integrated memo writing, quantification of relationship characteristics, and causal diagrams—visual depictions of possible relationships between different influences contributing to women’s experiences with nurses—as a means to refine understandings of the patient-provider relationship. Findings from transcripts, causal diagrams, and memos were triangulated to: 1) assess consistency across data sources, and 2) identify themes developing from discussions over time regarding data interpretation [22, 35]. The research team met every week for 3 months to complete the analysis process.

### Data representation

Interviewers and participants are designated with an ‘I’ (interviewer) and ‘P’ (participant) respectively. Each quote from a participant is labeled by her age and STI diagnosis at baseline, which is written using the respective acronym: *Chlamydia trachomatis* (CT), *Neisseria gonorrhoeae* (NG), and *Trichomonas vaginalis* (TV).

## Results

A total of 28 women were recruited to complete interviews, which lasted an average of 55 min. All participants were black African with a mean age of 28.7 years (range = 22–39 years), a median gestational age of 19 weeks (IQR = 11.5–23); 24 (86.7%) had 1 or more children already. Over half were unemployed, and > 90% had, at most, a high school diploma/matriculation degree. Regarding partner characteristics, 14 (50%) of women reported that their partner had another sexual partner, and 5 (18%) reported that they were unsure if their partner had another partner. A total of 12 women (42.9%) were diagnosed with HIV at their first antenatal care visit. By study completion, all interviewed women were successfully treated for STI’s and tested negative. Through the interviews, we found that women valued the nurses’ care and medical expertise. However, women didn’t trust the treatment immediately, and they demonstrated limited recall of their diagnosis and treatment.

### Role of counseling and support from nurses in promoting adherence to and trust in treatment

Supportive behavior from nurses was identified as important to participant’s treatment adherence and trust levels throughout the testing and treatment processes. Participants initially reported fear and guilt about their diagnoses: “*I felt like I let myself down. I don’t know how, but yah, I felt as if I let myself down by.. then I was scared for my baby*” (25-year old with CT & TV). One participant recalled upon receiving her diagnosis: “*I felt so lonely so sad sometimes I felt like ending my life but then I thought about my baby*” (28-year old with CT & NG). Support in the form of counseling at the time of diagnosis was identified by participants as being linked to stress reduction, confidence building, and skill building for disclosure to partners. Supportive behavior provided by nurses is described in the case below:

P: *She spoke to me like.. what can I say, like a friend. We were like speaking as [if we were] having a friendly conversation … A person speaking to you friendly, openly. You asking questions, or [them] asking you do you have any questions. It makes … me feel relieved …*. *Because if a person can give me the space to express what I feel or express what I do not understand about whatever situation I’m in like now. But, she gave me everything I would like [health information needed, and then] okay fine, I understand. Mm.*

I: *And, how did you feel after the counselling?*

P: *I felt free …* (25-year old with CT & TV)

Friendly nurses were described as those who allowed patients to ask questions without expressing judgment. Friendly care was differentiated from past negative care experiences: “*I was happy because I was getting help and the one who was helping me she gave me the support in everything … when you get to the clinic and you will find a person who will not judge and she will show you the way like some [other nurses] will judge and they would not show you what to do and what you mustn’t do*” (26-year old with TV). Nurses also helped women to feel confident in their ability to recover with treatment, helping one participant to: “*realize [the STI is] not [a] threatening disease it’s like you can accept it live with it longer and take treatment so that [it’s] not something you can be afraid of am not dying tomorrow or something you must have a positive mind*” (28-year old with CT & NG). Counseling provided by nurses regarding STI diagnoses helped patients to understand that proper treatment adherence would cure their infection.

Having developed a personal, trusting relationship with nurses additionally promoted adherence, along with symptom alleviation: “*because I trust the person who gave me the medication and when I was using them after two days I started seeing the differences and I was getting better*” (26-year old with TV). Supportive nurses helped women to feel confident disclosing their status to partners, as described by one participant:

I: *Ok, how did you feel about the counselling?*

P: *The counselling was good because it motivated me like to tell him [partner], because at first I was afraid that can I tell him or not? How will he react or something …*

I: *How did you feel before telling him?*

P: *Before telling him, I felt, like most of the time, [that] I was blaming myself thinking. That maybe it was because of me … I didn’t have a clear answer [explanation for how she acquired the STI] like, or it was a disappointment, but at the end, I realized that there was change in my life …*

I: *Ok, how did you feel after you told him this?*

P: *For me, it was a relief because I was holding something. That even if I don’t tell him, it’s going to haunt me. But, the minute I told him like everything, it was at ease.* (28-year old with CT & NG).

Supportive counseling by nurses at the time of diagnosis helped to alleviate initial anxiety that women expressed toward disclosing their diagnoses to their partners, and to have less fear surrounding their STI diagnoses in general. Supported women felt less guilt and more confidence when approaching their treatment plan.

### Influence of symptom reduction and negative re-test on trust of treatment over time

Despite trust in nurses and positive experiences with counseling, most participants did not immediately trust the treatment provided to them. This mistrust was grounded in hesitancy to take medication in general and uncertainty about the effectiveness of the STI treatments, which changed once their symptoms went away. Pill hesitancy was reflected in the case below:

*[On the] first day, I was like, ‘Are they crazy? Should I drink all these tablets? Are they insane? How am I going to do it?’ … because they gave me like … two [pills] of each the first time [for STI treatment] and then just one of the ARV’s, [and] so I was like, ‘How am I going to drink all these pills at once?’, because I wasn’t drinking any pills before. So now … everything has to change. I need to get used to drinking them, and some even has a smell. And then, sometimes you feel nausea [after taking the pills]; you want to vomit …* (28-year old with CT & NG).

In addition to this participant testing positive for two STIs, she was also diagnosed with HIV at her first ANC visit. As such, she was concerned about the number, size and smell of all the pills she was given. This participant’s concern was further exacerbated by the fact that she received multiple diagnoses at once. This was evident in her statement ‘everything has to change’ in her life—from taking no pills to taking many for STIs with regular HIV treatment. Many women acknowledged that their health changed significantly in one clinical visit, and that being diagnosed with HIV and an STI was a lot to understand and accept at once.

Once participants began their treatment, they were not certain that the treatment would work as explained to them by the nurse. Some stated that they would return to the clinic if ‘something goes wrong’. However, after being on their STI treatment for a few days, participants noticed their symptoms subsiding, as a participant stated: “*It was good because after two days, when I checked my underwear [before I tested for positive for STIs] I was feeling some smelly, but after two days that smell was gone”* (26-year old with TV). Many participants experienced increased trust in their medication after their symptoms had disappeared while on treatment. This trust was further bolstered when they received a negative STI result in their follow-up test-of-cure: “*Yes, because first it was positive then they gave us the medication then when I came back the second time it was negative so that was all”* (29-year old with CT).

In addition to bolstering trust, negative re-testing experiences provided closure. This could be seen both from the finality of the above participant’s wording of “*that was all*” (29-year old with CT) to the statement of another participant: “*they gave me pills and on the next visit told me am alright*” (24-year old with CT & NG). Receiving a final negative test result also reaffirmed positive experiences with the nurses and with the health care system, as shown in the below case study. The participant describes her experience with testing and treatment:

P: *… We [research nurse] ran a test [STI] … the result came positive. Then, they gave me treatment, [and] then I went home. On six weeks, they told me no … You are negative now. Then, I felt yo those people were so helping me, because I didn’t know that I have such … STIs in me. I didn’t know about those STI but, after participating in the study, I knew lot of STIs …*

I: *And how did you feel when you left the study and they said everything is okay?*

P: *I was so happy, because I was cured... Yes, they told me that now you are fine. You are cured. You don’t have that STI anymore*

(31-year old with CT)

This participant had two sequential STI-positive test results requiring her to complete two rounds of treatment. After the second round of treatment, the negative STI test result allowed the participant to see that the nurses were “so helping [her]” by teaching her about STIs and STI treatment, and by making sure that she was cured. She also expresses “I was so happy,” speaking to a sense of delight and relief that participants may have felt to leave their experiences with STIs behind them.

### Limited recall of medical information, an indicator of patient trust, may impact the ability to disclose to sexual partners

Although participants were able to speak clearly about their symptoms and experiences, most participants could not recall their exact diagnoses nor the treatment that they were given, stating, “*Yes, I forgot but they gave me tablets … I think it was Chlamydia and Gonorrhea something like that*” (29-year old with CT). The below case study is a good example of a typical exchange regarding the details that participants retained or had forgotten following the conclusion of their treatment:

*I: Can you remember the name of the STI?*

*P: No, I can’t. No, I don’t remember*

I*: What were your thoughts on your diagnosis?*

P*: Mm … because I wanted to protect my child you see … I didn’t want him or her to be affected [by the STI]. So that’s why I decided to take [the] test.*

I*: Do you think the infection is dangerous?*

P*: No, because it’s curable. It’s not that dangerous.*

I*: During your last clinic visit, can you tell me what medication you were given?*

P*: Mm..I was given pills to clean for [to treat the] STI that I was diagnosed with...*

*I don’t remember the name but only the pills.*

(26-year old with TV)

Participants remembered the parts of the STI experience that were important to them, such as their motivation to protect their child or the memory of taking the pills, but generally did not retain specifics as to the STI itself or the name of the medication. Rather, participants seemed to rely upon the nurse for this information and didn’t ask questions that could lead to ownership and recall of their diagnoses and treatments by making statements like, *‘Because sister [nurse] won’t give me medication that’s not working* …’ (29-year old with CT). This shift in focus to the nurses’ role may indicate that patient’s trust of the nurses’ expertise may diminish women’s engagement and focus to remember their diagnosis or treatment. Furthermore, this dependency on nurses along with limited recall of medical details likely impacted women’s ability to fully disclose to their partners. One participant stated, “*When [the nurse] was talking, I wished that my husband was here to listen because she talked so simple and you could understand [the diagnosis and treatment]*” (26-year old with TV). In expressing the desire for her partner to be present to listen to the nurse, this participant is acknowledging her own discomfort and uncertainty that she can explain the STI and treatment to her partner so that he “*could understand.*”

## Discussion

This qualitative study was designed to explore the experiences of pregnant women living with HIV regarding their communications with trained nurses while participating in a STI POC test and treat study. Our results reveal that nurses were able to establish and maintain trust with participants while engaged in the study. However, women had limited recall of their specific STI diagnosis and treatment, with implications for STI status disclosure to sexual partners and thus uptake of treatment by partners.

### Patient-centered care training may mitigate HIV and STI shame

We found that recurring, patient-centered care fostered a positive STI testing and treatment experience for pregnant women living with HIV, with no descriptions of shame associated with being pregnant while living with HIV [14, 36]. Often, pregnant women concurrently diagnosed with a STI and HIV are stigmatized by nurses for their infection and the assumed risky sexual behaviors associated with their infection(s) [37]. Our study suggests that nurse training in compassionate care and motivational interviewing, as components of a comprehensive POC STI test and treat intervention, helped women to mitigate feelings of guilt and shame, allowing some of them to disclose their STI status to their partners and more confidently approach their treatment [20, 38]. Furthermore, the care participants received from study nurses was described as non-judgmental compared to their previous care experiences. Finally, most women understood the implications of their diagnoses, their capacity to recover, and motivation to complete treatment and disclose their STI diagnosis to their partner(s) as a way to protect their unborn child [8].

### Interpretation of limited recall around STI diagnosis and treatment details

A positive patient-provider relationship did not appear to enhance women’s medical understanding of their STI nor their treatment [21]. When recounting their treatment experiences, participants spoke almost exclusively of the emotional support they received during counseling, but did not discuss the medical information they were given about their STIs. Such a tendency may indicate a limited recall or understanding of their diagnosis of treatment. This apparent contradiction may shed light on how women of lower socioeconomic background, poor health literacy or lower educational attainment move through the health care system [39, 21]. Furthermore, it is likely that some of these factors inherently interact with each other. For instance, an educational background ending in secondary school may not delve into science as thoroughly as in higher education, which influences health literacy and ability to understand medical terminology [40, 41]. Limited health literacy in turn may affect the ability to ask the right questions or absorb basic information their providers are providing them [42]. This disconnect may create a gap in patient-provider communication, where the provider feels they have given the patient the necessary information, but the patient may not actually have received it [43]. Ultimately, without a proper understanding of what STI they were diagnosed with and what treatment they are taking, individuals may struggle to explain to their partners the urgency to seeking care, the treatment they are taking or the necessary steps to prevent reinfection.

### Study limitations

Our study is limited by the lack of interviews conducted with women who had a negative test result. As such, we cannot assess the role of trust and health literacy on receiving a negative test result. This could have major implications if a woman were to present with an incident STI later in pregnancy. Another study limitation is that we did not assess a women’s true level of health literacy. As such, we may not have clear enough insight on how to further improve the provision of health information provided in our current intervention. Consequently, additional research is needed to understand what basic level of health literacy is needed to maximize trust in point-of-care testing during pregnancy.

## Conclusions

South African pregnant women living with HIV trusted nurse treatment of their STI infection in this study. Given how participants described patient-centered care characteristics of study nurses, these results are likely a result of the specialized nurse training we provided in motivational interviewing, compassionate care and good clinical practice. However, participants had limited recall of their diagnosis and treatment. This lack of recall may pose significant barriers to disclosure and care self-management. Furthermore, most women described an over-reliance on nurses, which may be reflect limited health literacy among women and men in this setting. Of note, we have reported that ~ 80% of women in our larger study with an STI returned for their test-of-cure visit [44]. While this represents a high level of study retention, the experiences of women that did not return, and thus could not be interviewed, may be qualitatively different from women that were interviewed.

As POC diagnostics for STIs are scaled up, ensuring their maximal public health impact will require more than just optimal testing coverage. Towards this, when an individual discloses their STI status to their sexual partner(s) and those partners seek treatment, the risk of re-infection can be strongly diminished. Conversely, any barriers to STI status disclosure can have a direct impact on partner treatment uptake, and thus re-infection risk. To ensure that POC STI diagnostic testing interventions have the desired public health effect, a comprehensive POC intervention package that includes improved health literacy, partner notification support and counselling and partner treatment uptake, must be implemented.

## Data Availability

All the data supporting our findings are contained within this manuscript. Datasets used in this study may be requested from corresponding author: Dr. Andrew Medina-Marino (email: andrewmedinamarino@gmail.com).

## Abbreviations

STI: Sexually transmitted infections
POC: Point-of-care
CT: *Chlamydia trachomatis*
TV: *Trichomonas vaginalis*
NG: *Neisseria gonorrhea*
ANC: Antenatal care
PMTCT: Prevention of mother to child transmission
ART: Antiretroviral therapy
TOC: Test-of-cure
HIV: Human immunodeficiency virus
IDI: In-depth interview
IQR: Interquartile range
